# Kresling Origami‐Based Metamaterial Robot for Dynamic Electromagnetic Control

**DOI:** 10.1002/advs.76437

**Published:** 2026-07-17

**Authors:** Xinxi Zeng, Long Zhou, Shaojie Xi, Yixuan Gao, Desheng Pan, Chaoyang Sun, Haoxin Sun, Xian Wang, Lingyun Qian, Peipei Li, Pengfei Zhu, Bo Li, Jiang Luo, Ji Zhou

**Affiliations:** ^1^ School of Mechanical Engineering University of Science and Technology Beijing Beijing People's Republic of China; ^2^ State Key Laboratory of New Ceramic Materials School of Materials Science and Engineering Tsinghua University Beijing People's Republic of China; ^3^ Institute of Materials Research Tsinghua Shenzhen International Graduate School Tsinghua University Shenzhen People's Republic of China; ^4^ Beijing Bayi School International Department Beijing People's Republic of China; ^5^ State Key Laboratory for Advanced Metals and Materials University of Science and Technology Beijing Beijing People's Republic of China; ^6^ The Ninth Research Institute of China Electronics Technology Group Corporation Mianyang People's Republic of China; ^7^ Foshan (Southern China) Institute for New Materials Foshan People's Republic of China; ^8^ School of Electronics and Information Hangzhou Dianzi University Hangzhou People's Republic of China

**Keywords:** active modulation, Kresling origami, metamaterial, robotic system

## Abstract

Reconfigurable metamaterials have enabled significant progress toward tunable microwave devices, yet existing approaches are often hindered by limited mechanical reconfigurability and susceptibility to electromagnetic interference. Here, we introduce a type of metamaterial robot that harnesses bistable Kresling origami to achieve dynamic electromagnetic control. By integrating pneumatically driven folding architectures with digital light processing–printed SrTiO_3_ high‐permittivity ceramic patches, our design establishes a unified platform where mechanical deformation and microwave resonant behavior can be actively and synergistically regulated. The coupled extension‐twisting motion inherent to the Kresling geometry simultaneously tailors the axial height, in‐plane orientation, local propagation phase, and boundary conditions of the ceramic resonators, thereby modulating the excitation conditions of dielectric resonance responses across distinct frequency bands. Experimentally, transitioning a single origami unit from its folded state to its deployed state increases the number of resolvable reflection peaks from two to three, with a minimum reflectance reaching approximately −35.6 dB. Rotating the SrTiO_3_ patches further shifts the dual‐resonance center frequencies from 3.71–3.87 Hz and 16.72–17.26 GHz to 4.36–4.45 GHz and 16.31–16.72 GHz, respectively. Expanding this concept to a spatially heterogeneous origami array enables coupled control of both resonance peaks, yielding a minimum peak reflectance as low as −54.4 dB and a reflection modulation depth of 51.0 dB at 12 GHz.

## Graphical abstract

A reconfigurable metamaterial system integrates deformable origami units with high‐permittivity ceramic plates. Pneumatic switching between structural states alters local resonance conditions, enabling dynamic radar stealth and adaptive microwave reflection for mobile communication.

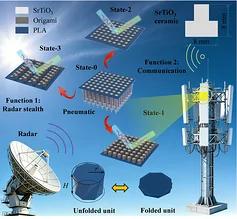

## 1 Introduction

Metamaterials have emerged as a powerful platform for manipulating electromagnetic waves through subwavelength‐scale structuring of matter [1, 2]. By carefully tailoring the geometry, composition, and spatial arrangement of unit cells, these artificial media offer precise control over electromagnetic waves [3, 4]. A fundamental limitation, however, plagues conventional metamaterials: once fabricated, both their geometry and their electromagnetic functionality remain permanently locked. To overcome this rigidity, various tuning strategies have been explored, including phase‐change materials, liquid crystals, and optically controlled metasurfaces [5, 6]. Despite their individual merits, these approaches often introduce substantial system‐level complexity without fully decoupling reconfigurability from the actuation infrastructure. Most demand continuous power input, auxiliary biasing networks, or specific external field conditions, and many remain inherently susceptible to electromagnetic interference that undermines their reliability in realistic microwave environments.

An alternative and conceptually distinct pathway lies in mechanical reconfiguration [3, 7]. By directly altering the positions, orientations, and mutual coupling conditions of resonant elements via structural deformation, this strategy achieves spectral tuning through geometry rather than through changes in material state. Among mechanical schemes, origami‐inspired architectures have attracted considerable attention owing to their programmable folding kinematics, large deformation range, and reversible switching between stable configurations [7, 8]. In particular, Kresling origami, featured with intrinsic coupled extension‐twist motion and excellent multistable characteristics, has been extensively investigated for mechanical vibration control and energy harvesting in recent years. Relevant studies have verified that Kresling structures can construct effective vibration bandgaps and serve as high‐performance vibration isolators, while their unique deformation modes also enable efficient mechanical energy transduction and collection [9, 10, 11, 12]. Nevertheless, the integration of origami with high‐permittivity ceramic patches for dynamic control of microwave dielectric resonances remains largely uncharted territory. Existing origami‐based electromagnetic systems have predominantly relied on global shape changes or single‐configuration transitions, leaving the untapped potential of multiunit coordination, spatially heterogeneous arrangements, and colocalized actuation control largely unexploited. A persistent challenge therefore remains: simultaneously achieving large reversible deformation, stable bistable configuration retention, and compact integration of dielectric resonators [9].

Herein, we introduce a Kresling origami‐based metamaterial robot that directly confronts these challenges. The proposed platform synergistically integrates bistable folding architectures, digital light processing‐printed SrTiO_3_ ceramic patches, and pneumatic actuation into a unified reconfigurable assembly. Central to our design is the exploitation of the coupled extension‐twisting motion inherent to each Kresling unit, which concurrently tailors the axial height, in‐plane orientation, local propagation phase, and boundary conditions of the ceramic resonators [13, 14]. This geometry‐driven paradigm enables structure‐mediated reconfiguration of the microwave response without relying on electronic components, sustained external fields, or continuous power supply—a distinct advantage over existing tuning methods. Characterizing a single origami unit experimentally reveals that transitioning from the folded state to the deployed state increases the number of resolvable reflection peaks from two to three, with the minimum reflectance reaching approximately −35.6 dB. Moreover, rotating the SrTiO_3_ patches shifts the dual‐resonance center frequencies from the ranges of 3.71–3.87 GHz and 16.72–17.26 GHz to 4.36–4.45 GHz and 16.31–16.72 GHz, respectively. When this concept is extended to a spatially heterogeneous origami array, coupled control over both resonance peaks becomes attainable, yielding a minimum peak reflectance as low as −54.4 dB and a reflection modulation depth of 51.0 dB at 12 GHz. Collectively, these findings establish a Kresling‐origami‐driven paradigm for electromagnetic response regulation, opening a new structural route toward programmable and reconfigurable microwave devices.

## 2 Experimental Section

The dielectric ceramic components were fabricated from SrTiO_3_ powder, 1,6‐hexanediol diacrylate (HDDA), and polyethylene glycol diacrylate (PEGDA). Diphenyl (2,4,6‐trimethylbenzoyl) phosphine oxide (TPO) was used as the photoinitiator, and KOS110 acted as a dispersant. HDDA, PEGDA, and SrTiO_3_ were mixed at a weight ratio of 1:1:9. Subsequently, KOS110 (5.6 wt.% relative to SrTiO_3_) and TPO (3 wt.% relative to the resin) were added to the mixture. The mixture was then ground in a planetary ball mill at 350 rpm for 3 h to obtain a homogeneous photosensitive SrTiO_3_ slurry with a solid content of ∼78 vol%. A DLP‐based ceramic 3D printer was then used to fabricate the T‐shaped green bodies. The green bodies were subsequently sintered in a muffle furnace under an air atmosphere. The sintering process involved heating to 1300°C at a rate of 5°C/min, followed by a 3 h dwell time, resulting in the formation of SrTiO_3_ ceramic sheets.

Mechanical testing was conducted using a microcomputer‐controlled electronic universal testing machine (CMT4103, MTS, USA). The machine has a maximum load capacity of 1 kN and complies with accuracy class 0.5 (resolution: 0.001 N). Uniaxial compression tests on the Kresling origami units were conducted using a custom‐designed bearing fixture at a displacement rate of 3 mm/min at room temperature.

The reflectance of the metamaterials was measured using an arch measurement method integrated within an electromagnetic material testing system (CETC Siyi Technology Co., Ltd.). The system comprises a vector network analyzer, an arch frame fixture, and broadband horn antennas covering the 2–18 GHz frequency range. Before testing, a metal plate was placed on the sample holder for calibration. Afterward, the test sample was mounted on the holder to measure the reflectance. The included angle between the incident and reflected beams was set to 10° throughout the measurement.

Pneumatic experiments were performed using a device based on Kresling origami. The overall elongation and contraction of the structure were achieved by the extension and compression of syringes. An oil‐free air compressor provided stable air pressure, and a pressure regulator (IR2000‐02BG) controlled the pressure inside the syringe chamber, enabling controllable actuation of the motion.

## 3 Results and Discussion

The proposed system integrates deformable origami units and high‐permittivity T‐shaped SrTiO_3_ ceramic plates on a robust PLA array substrate, forming an integrated reconfigurable platform for structural deformation and electromagnetic response modulation. As illustrated in Figure 1, the system shows potential utility for radar protection and mobile communication. Starting from the initial configuration, State‐0, the metamaterial system can be actuated into different structural states, including State‐1, State‐2, and State‐3, according to external electromagnetic requirements. Owing to the extension‐torsion coupled deformation of the origami units, the relative spatial positions of the T‐shaped SrTiO_3_ ceramic plates are reconfigured, thereby altering the local resonance boundary conditions of the overall metasurface. Under multiband radar detection conditions, the structure can be pneumatically switched to the low‐reflection or low‐scattering state represented by State‐3, offering a potential approach for dynamic radar stealth and electromagnetic camouflage. When interaction with communication base stations is required, the structure can be transformed into the configuration represented by State‐1 to reconstruct the electromagnetic response of the surface and modulate microwave reflection in specific frequency bands, thereby adapting to different communication requirements.

**FIGURE 1:**
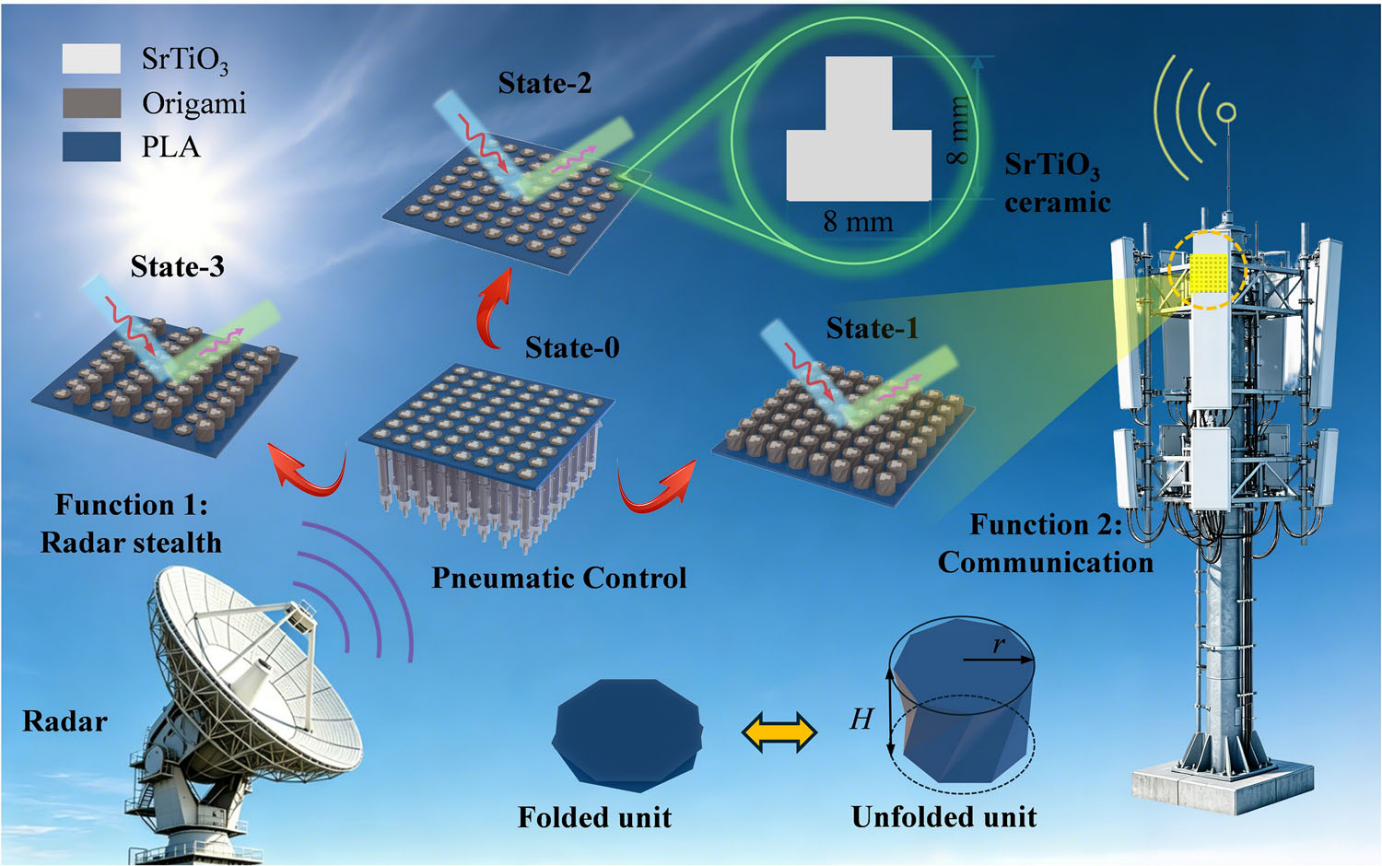
Potential application scenarios of the multifunctional reconfigurable metamaterial system.

### 3.1 Geometric and Mechanical Models

The Kresling origami unit was used as the core deformation‐regulating element of the metamaterial robot, and a coupled model was established based on Figure 2 to provide a structural basis for electromagnetic‐wave modulation through robotic deformation. Figure 2a–d shows the geometric characteristics of the Kresling origami unit in the fully unfolded state and fully folded state. The green regions denote the upper and lower regular polygonal surfaces, the blue lines denote the mountain creases *L*_S_, and the red lines denote the valley creases *L*_G_. Two assumptions were adopted in the model: the upper and lower surfaces remain parallel with unchanged geometric parameters, and the length of *L*_G_ remains constant during folding. These assumptions ensure controllable deformation of the robotic unit. The extension‐torsion coupled motion of the Kresling origami, which serves as the core deformation mode of the robot, determines the posture‐tuning capability of the ceramic plates. Its bistability is evaluated under the condition that the strain of the mountain crease *L*_S_ is zero in both the unfolded and folded states.

**FIGURE 2:**
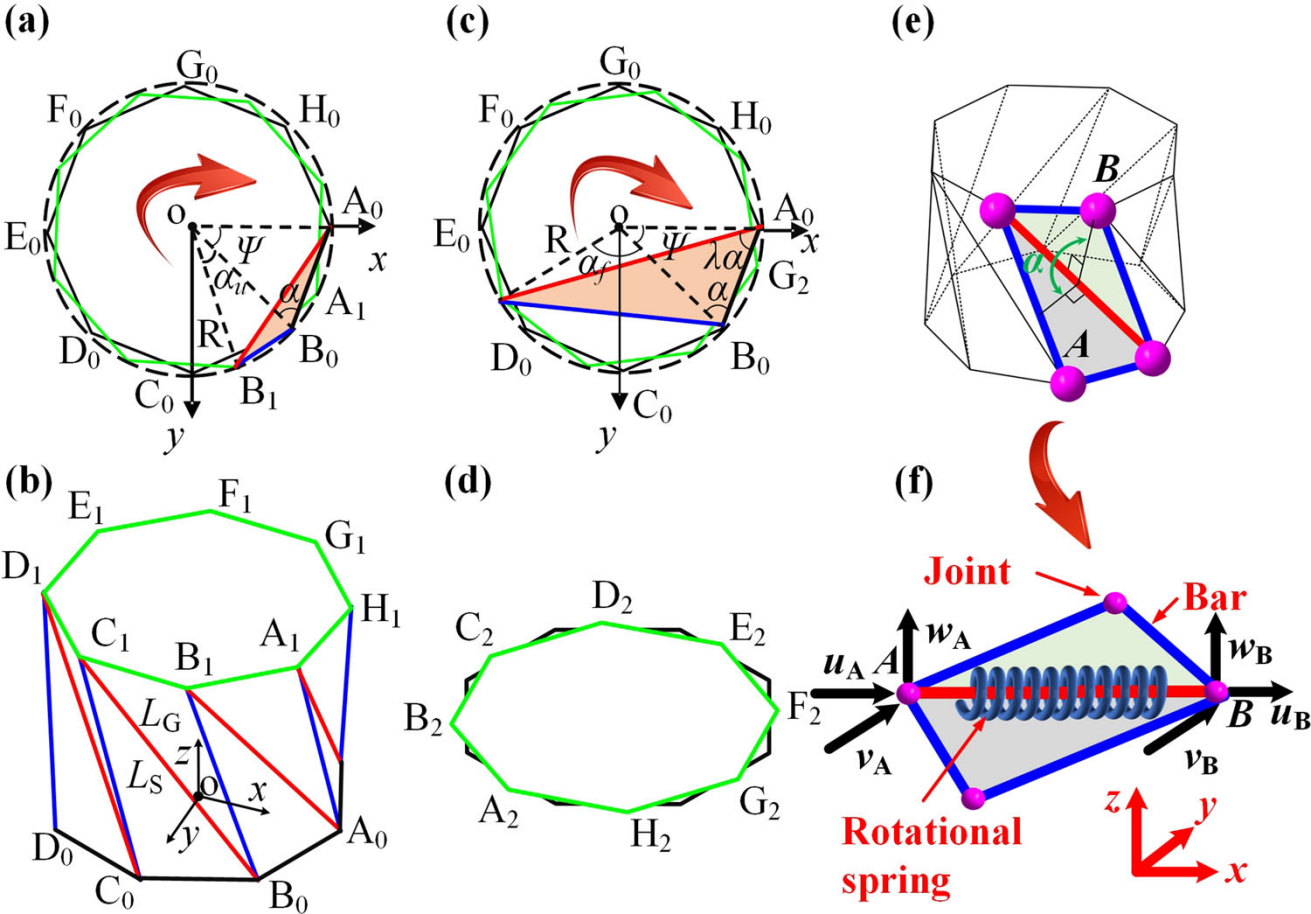
Geometric and mechanical models of Kresling origami: (a,b) Unfolded state; (c,d) Folded state; (e) Simplified truss model [16, 17, 18]; (f) Fundamental model of folded surface composed of rods, hinges, and torsional springs [19, 20].

Figure 2e,f shows the robotic driving mechanical model established based on the stationary potential energy principle [15]:

(1) $$\xi =U_{bar}+U_{spring}-W_{out}$$

(2) $$\frac{\partial \xi }{\partial u}=\frac{\partial U_{bar}}{\partial u}+\frac{\partial U_{spring}}{\partial u}-F_{out}=F_{bar}+F_{spring}-F_{out}=0$$

(3) $$K_{bar}+K_{spring}=K$$

Here, ξ is the total potential energy of the system, *W*_out_ is the work done by the external load, *U*_bar_ is the rod strain energy, and *U*_spring_ is the rotational spring strain energy. *F_bar_* is the internal force in the rod, *F*_spring_ is the internal force in the rotational spring, *F*_out_ is the external load on the rod node, and *u* is the nodal displacement vector. *K*_bar_ is the tangential stiffness of the rod, *K*_spring_ is the tangential stiffness of the rotational spring, and *K* is the overall system tangential stiffness.

The fabrication process of the Kresling origami unit used in the experiment is shown in Figure 3a, and the customized fixture with a rotating bearing is shown in Figure 3b. Figure 3c–e shows the extension‐torsion coupled motion of the origami unit. During compression, the unit sequentially undergoes elastic, folding, and stable stages. The experimental curves in Figure 3f,g show that a larger planar angle fraction *λ* leads to a higher maximum output force and stiffness. When *λ* = 0.92, the maximum output force is approximately 1.6 N, which is 2.7 times that obtained at *λ* = 0.78. A lower *λ* corresponds to lower stiffness, which is more favorable for posture adjustment of the ceramic plates through large deformation and ultimately supports precise modulation of microwave reflection characteristics. The stress distribution during compression is shown in Figure 3h. During compression, the stress on the connecting faces between creases is much lower than that on the creases, indicating that the creases are the primary load‐bearing components of the origami structure.

**FIGURE 3:**
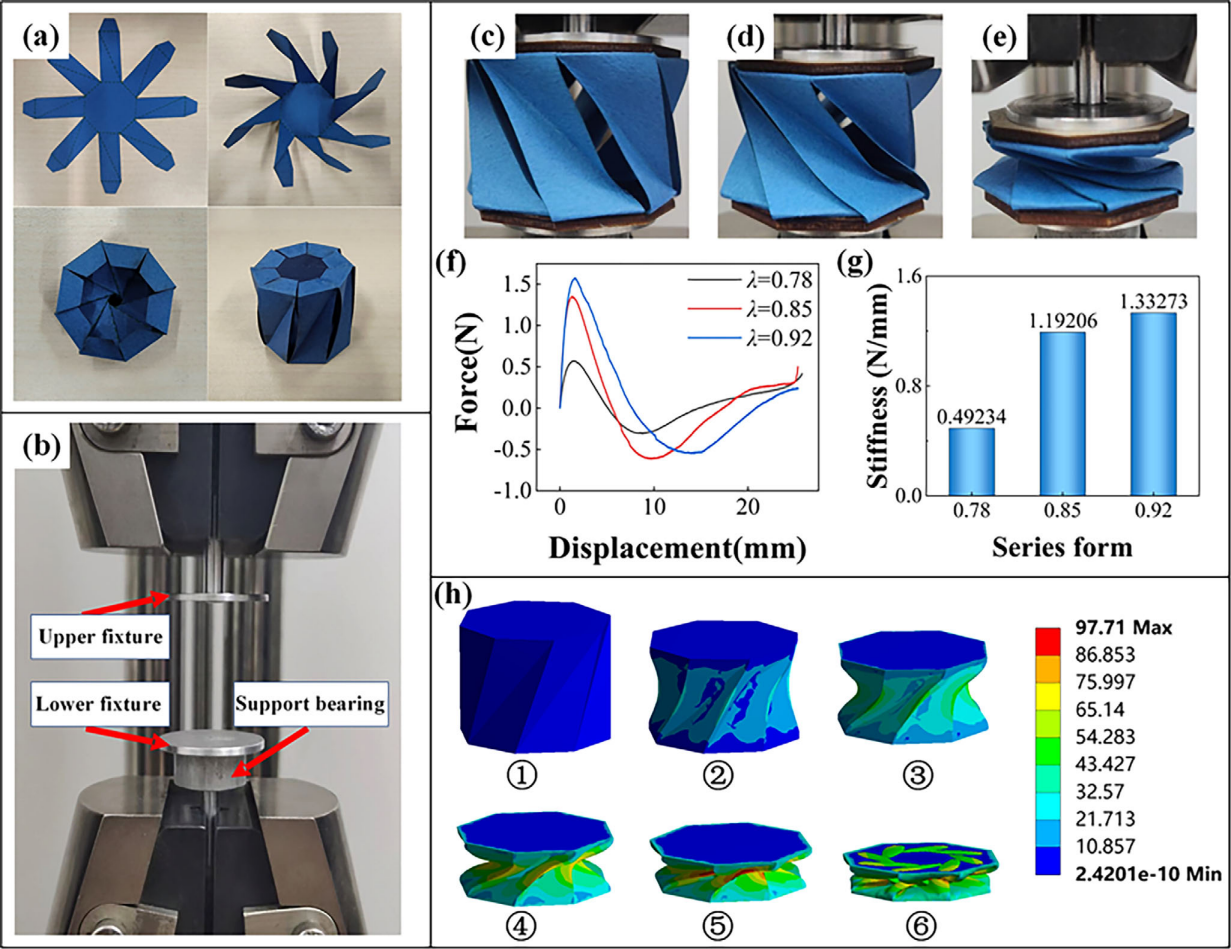
(a) Folding process; (b) compression testing setup; (c–e) elastic stage, folding stage, and stable stage; (f) effect of geometric parameter λ on the deformation of the origami unit; (g) effect of geometric parameter λ on the mechanical properties of the origami unit; (h) stress distribution of the origami structure during compression.

### 3.2 Experimental Setup and Structural Design

As shown in Figure 4a, time‐domain gating was used in the microwave anechoic chamber to suppress environmental stray reflections [21]. The reflectance was measured using the arch method, with the angle between the incident and reflected beams set to 10°. When 0.5 < *λ* ≤ 1, the Kresling origami unit exhibits bistability, whereas the number of sides n of the regular polygon does not affect the stable states of the origami unit. Therefore, an origami unit with *λ* = 0.78, *n* = 8, and *R* = 15 mm was selected. The folded and unfolded states of the origami are shown in Figure 4b,c, respectively.

**FIGURE 4:**
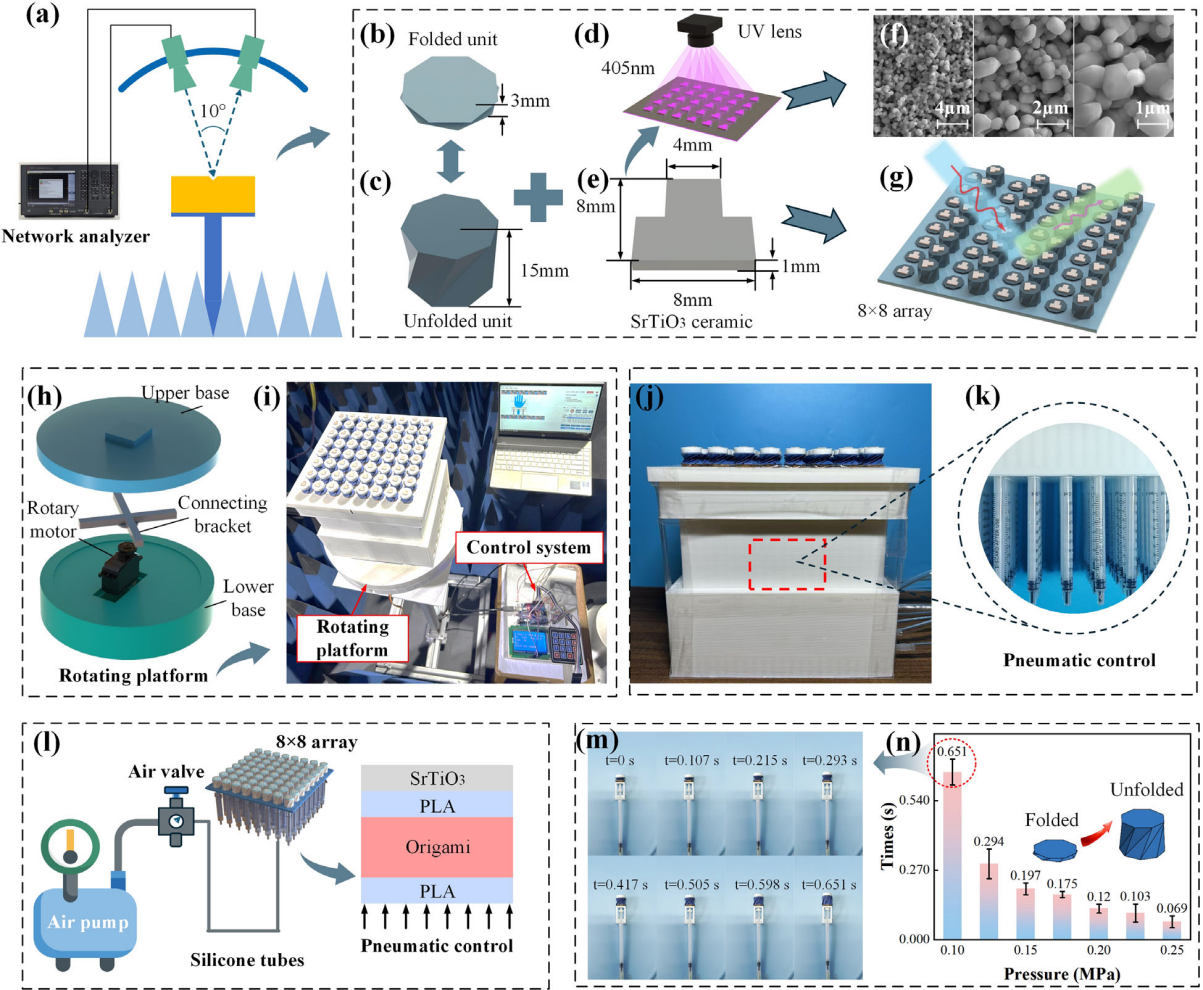
(a) Principle of reflectance measurement using the arch method; (b,c) folded and unfolded states of the Kresling origami; (d) principle of DLP‐based 3D printing of SrTiO_3_ ceramics; (e) T‐shaped SrTiO_3_ ceramic plate; (f) SEM image of SrTiO_3_ after sintering at 1300°C; (g) 8 × 8 electromagnetic unit array; (h) structural composition of the rotation platform; (i) rotation platform and control system; (j) Kresling origami robot; (k) syringe‐based pneumatic actuation array; (l) composition of the pneumatic actuation system; (m) switching process of a single pneumatic unit from the folded state to the unfolded state at 0.10 MPa, the minimum pressure required for configuration switching; (n) switching time of a single pneumatic unit from the folded state to the unfolded state under different pressures from 0.10 to 0.25 MPa.

The T‐shaped SrTiO_3_ ceramic plate shown in Figure 4e was fabricated using the DLP‐based ceramic 3D printing method shown in Figure 4d. The wavelength of the ultraviolet light was 405 nm. The ceramic plates were sintered at 1300°C, and the corresponding scanning electron microscopy image is shown in Figure 4f. The dimensions of the T‐shaped SrTiO_3_ ceramic plate were determined by considering the electromagnetic response, structural compatibility, and mechanical load. Previous studies on high‐permittivity ceramic all‐dielectric metamaterials have shown that the geometry, dimensional parameters, and dielectric constant of ceramic resonators are key factors determining their microwave resonance responses [22, 23]. The ceramic unit used in this study has an overall circumscribed dimension of 8 × 8 mm and a thickness of 1 mm. Within the 2–18 GHz testing band, the free‐space normalized electrical size of the 8 mm lateral dimension, defined as L/Lλ0λ0, is approximately 0.053–0.48, where *L* = 8 mm is the lateral size of the ceramic pattern, and *λ*_0_ is the free‐space wavelength. In the low‐frequency response region of 3–4 GHz, the 8 mm ceramic pattern size is approximately 0.088–0.110 times the corresponding free‐space wavelength, indicating that the ceramic pattern has a compact subwavelength feature in the low‐frequency range. In the mid‐to‐high‐frequency region, the electrical size corresponding to 8 mm gradually increases with frequency, which can enhance the local interaction between the high‐permittivity ceramic plate and the incident microwave.

Structurally, the Kresling origami unit has a circumscribed radius of *R* = 15 mm, and the T‐shaped SrTiO_3_ ceramic plate can be placed in the central region of the unit while maintaining good structural compatibility with crease motion and extension‐torsion coupled deformation. Mechanically, the mass of a single ceramic plate is approximately 0.15–0.16 g, allowing the origami unit to maintain stable bistable behavior. As shown in Figure 4g, a T‐shaped SrTiO_3_ ceramic plate was attached to the top of each Kresling origami unit to form an 8 × 8 unit array for investigating electromagnetic response characteristics.

To investigate the electromagnetic response under different rotation angles, a rotation platform was designed, as shown in Figure 4h. The platform integrates a stepper motor and a gear transmission system, and the rotation angle is controlled by an Arduino UNO‐R3 microcontroller, as shown in Figure 4i. Pneumatic actuation was used to dynamically modulate the electromagnetic response of the Kresling‐origami‐based metamaterial, and the experimental setup is shown in Figure 4j,k. As shown in Figure 4l, the system is driven by a syringe‐based pneumatic actuation unit, in which an air pump, air valve, and silicone tubes form a pneumatic network that converts air‐pressure signals into coupled changes in the height and torsion angle of the origami units. Specifically, the air pressure generated by the pump is regulated by the air valve and transmitted to the bottom chamber of the syringe through silicone tubes, driving the piston rod upward. The top of the piston rod is connected to the center of the upper surface of the origami through a plastic bearing, whereas the lower surface of the origami is fixed to the base plate, forming a deformable structure capable of axial extension and rotation.

When the air pressure exceeds the critical value for configuration switching, the unit transforms from the folded state to the unfolded state, and the reverse transition can also be achieved. This fully mechanical actuation strategy helps reduce electromagnetic interference from electrically controlled components and improves the reliability of reflection measurements in the microwave anechoic chamber. As shown in Figure 4m,n, the switching time of a single origami unit from the fully folded state to the fully unfolded state was measured under different air pressures. At 0.10 MPa, the minimum pressure required for configuration switching, the switching time is approximately 0.651 s. At 0.25 MPa, the switching time decreases to approximately 0.069 s. After switching, the electromagnetic response stabilizes within approximately 0.1 s, indicating a subsecond dynamic response. Although the pneumatic system responds more slowly than electrically controlled systems, the Kresling origami robot used in this study can still achieve subsecond state switching and electromagnetic‐response stabilization by increasing the driving pressure. This response speed is sufficient for most quasi‐static tuning and dynamic reconfiguration scenarios in microwave modulation.

### 3.3 Electromagnetic Wave Manipulation

The T‐shaped SrTiO_3_ ceramic plate has high‐permittivity characteristics and a non‐axisymmetric geometry, enabling the formation of a localized dielectric resonance response in the microwave regime. Unlike conventional metamaterials with fixed structures, the T‐shaped SrTiO_3_ ceramic plate in this work is attached to the upper surface of the Kresling origami unit, so that its spatial posture changes synchronously with the axial extension and in‐plane rotation of the origami unit. According to the geometric model described in Section 3.1, the transition of Kresling origami from the folded state to the unfolded state is accompanied by axial height variation and net end‐surface rotation. Consequently, the vertical position, in‐plane orientation, and propagation phase condition of the ceramic plate relative to the incident and reflected electromagnetic waves are altered, leading to the reconstruction of the local electric‐field distribution and polarization‐current pathway.

To describe the extension‐torsion coupled relationship of the Kresling origami unit during the folded‐to‐unfolded transition, its upper and lower end surfaces are simplified as two congruent regular n‐gons with a radius of *R*. The half‐angle parameter corresponding to two adjacent vertices on the end surface is defined as *α*. The planar angle fraction *λ* is used to determine the end‐surface rotation angle *α_s_* of the two end states of the Kresling unit and further determines its bistable geometric characteristics [24, 25], where *s* denotes the state of the Kresling unit, and *f* and *u* correspond to the folded and unfolded states, respectively. In the folded and unfolded states, the rotation angles of the upper end surface relative to the lower end surface are given by

(4) $$\alpha =\left(\pi -\psi \right)/2,\alpha_{f}=\lambda \left(\pi -\psi \right),\alpha_{u}=\left(1-\lambda \right)\left(\pi -\psi \right)$$

Thus, the net rotation angle from the folded state to the unfolded state can be expressed as

(5) $$\Delta \alpha =\left|2\lambda -1\right|\left(\pi -\psi \right)$$

The valley‐crease length *L*_G_ remains constant during the folded‐to‐unfolded transition. According to the geometric relationship of the valley crease, the axial height under the rotation angle *α*_s_ can be written as

(6) $$H\left(\alpha_{s}\right)=\sqrt{L_{G}^{2}-2R^{2}\left[1-\cos(\alpha_{s}+\psi )\right]}$$

Accordingly, the axial height variation from the folded state to the unfolded state is expressed as

(7) $$\Delta H=H\left[\left(1-\lambda \right)\left(\pi -\psi \right)\right]-H\left[\lambda \left(\pi -\psi \right)\right]$$

The above equations indicate that the folded‐to‐unfolded transition of Kresling origami is essentially an extension‐torsion coupled motion involving both axial height variation and end‐surface rotation [26, 27]. This coupled motion is jointly determined by the number of sides of the end surface n, the circumscribed radius *R*, the valley‐crease length *L*_G_, and the planar angle fraction *λ*. Specifically, the net rotation angle Δ*α* characterizes the change in the in‐plane orientation of the upper end surface during the folded‐to‐unfolded transition, whereas the axial height variation Δ*H* characterizes the corresponding vertical displacement. Together, these two quantities constitute the primary geometric outputs of the Kresling origami during the transition from the folded state to the unfolded state and provide the structural basis for analyzing the spatial posture variation and electromagnetic resonance modulation of the T‐shaped SrTiO_3_ ceramic plate.

The axial height variation modifies the spatial position of the ceramic plate relative to the incident and reflected waves and introduces a propagation phase variation. For the arch‐method reflectance measurement, when the incident and receiving angles are denoted as *θ*_i_ and *θ*_r_, respectively, the propagation phase variation induced by the height variation Δ*H* can be expressed as

(8) $$\Delta \phi_{H}=k_{0}\Delta H\left(\cos\theta_{i}+\cos\theta_{r}\right)$$

where *k*_0_ = 2π/*λ*_0_ is the free‐space wavenumber and *λ*_0_ is the free‐space wavelength. Equation (8) indicates that the axial extension of the Kresling origami can modify the phase relationship and local boundary condition between the ceramic resonant units and the incident and reflected electromagnetic waves [28]. For high‐permittivity T‐shaped SrTiO_3_ ceramic plates, the dielectric resonance response originates from the polarization response and polarization‐current distribution excited inside the ceramic by the incident electric field [29, 30]. Under the local linear and spatially isotropic dielectric approximation, the polarization intensity at the center frequency $f_{q}^{\left(s\right)}$ of the *q*‐th resonance peak in state *s* can be expressed as

(9)

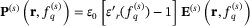

where $E^{\left(s\right)}\left(r,f_{q}^{\left(s\right)}\right)$ is the local electric‐field distribution obtained from the CST E‐field monitor at the center frequency of the corresponding resonance peak in state *s*, *ε*_0_ is the vacuum permittivity, and 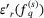 is the real part of the relative permittivity of SrTiO_3_ at this frequency. The time‐varying polarization generates a polarization‐current density, which can be written as

(10) $$J_{p}^{\left(s\right)}\left(r,f_{q}^{\left(s\right)}\right)=j\;2\pi f_{q}^{\left(s\right)}P^{\left(s\right)}\left(r,f_{q}^{\left(s\right)}\right)$$

Equations (9) and (10) indicate that the polarization response and polarization‐current pathway inside the ceramic plate are jointly determined by the local electric‐field distribution and the center frequency of the corresponding resonance peak [31]. Therefore, the modulation of the electromagnetic response induced by Kresling origami deformation can be attributed to the reconfiguration of the ceramic plate position, in‐plane orientation, propagation phase condition, and local boundary condition during the folded‐to‐unfolded transition. This reconfiguration alters the excitation conditions of dielectric resonance responses in different frequency bands and is ultimately manifested as variations in the number of resonance peaks, center frequency, and reflection‐valley depth. Therefore, the additional mid‐frequency resonance peak observed in the unfolded state is attributed to the reconstruction of the local dielectric resonance response induced by Kresling deformation.

As shown in Figure 5a, the Kresling origami structure can switch between the fully folded state State‐0 and the fully unfolded state State‐1. Figure 5b,c shows the three‐dimensional mapping results of reflectance as a function of frequency and rotation angle under the two configurations, respectively. In State‐0, the sample mainly exhibits two resolvable resonance peaks; after switching to State‐1, three resolvable resonance peaks appear in the reflection spectrum. Figure 5d–f further shows the reflectance spectra in representative frequency bands, indicating that after origami unfolding, the low‐frequency peak undergoes a redshift, a new resolvable reflection valley appears near 9.97 GHz in the mid‐frequency region, and the high‐frequency peak undergoes a blueshift. These results indicate that Kresling configuration switching can modulate dielectric resonance responses in different frequency bands. Figure 5g–k shows the center frequencies of different resonance peaks as functions of the rotation angle *θ*, where the red dotted lines in Figure 5h–k represent the CST simulation results and the black dotted lines represent the experimental results. The two results are generally consistent in terms of peak‐position variation and angle‐dependent trends. Specifically, in State‐0, the low‐frequency peak varies only slightly over most rotation angles, whereas the high‐frequency peak exhibits more pronounced angle dependence. In State‐1, the low‐frequency and mid‐frequency peaks remain within relatively stable ranges of approximately 3.24–3.30 GHz and 9.96–10.05 GHz, respectively, while the high‐frequency peak shows more evident frequency fluctuations with the rotation angle, indicating that the high‐frequency response is more sensitive to the in‐plane orientation of the ceramic plates. Figure 5l–p shows the reflectance amplitudes of different resonance peaks as functions of the rotation angle. In State‐0, the reflection‐valley depths of the two peaks exhibit similar variation trends with the rotation angle. In State‐1, the amplitude modulation of the low‐frequency and mid‐frequency peaks is relatively limited, whereas the high‐frequency peak exhibits a larger reflectance modulation range. These results indicate that the extension‐torsion coupled deformation of the Kresling origami can modulate the number of resonance peaks, center frequencies, and reflection‐valley depths in the reflection spectrum by changing the axial height, in‐plane orientation, and local boundary conditions of the T‐shaped SrTiO_3_ ceramic plates. Under a fixed configuration, overall rotation of the array further introduces angle‐dependent electromagnetic response modulation.

**FIGURE 5:**
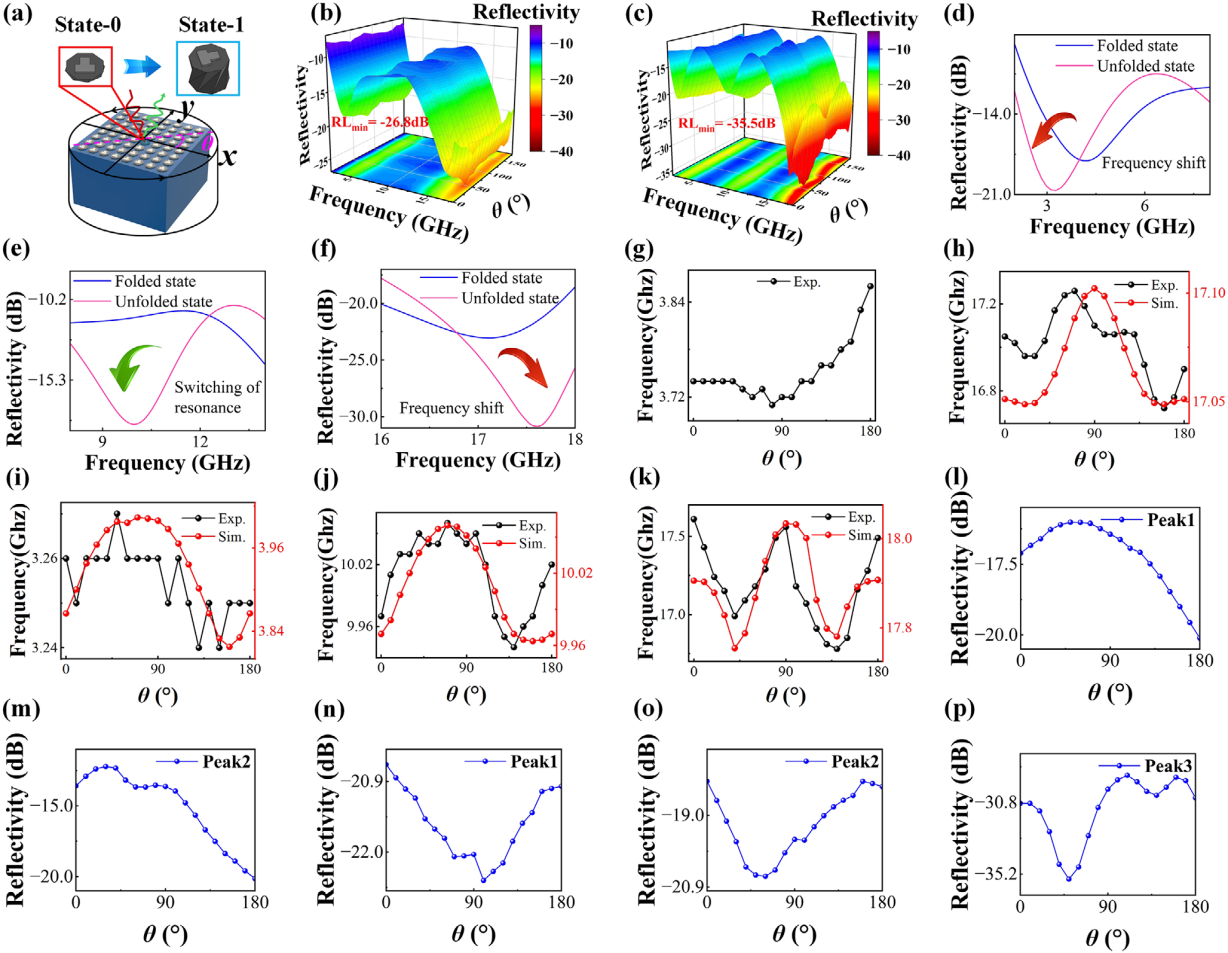
(a) Fully folded state (State‐0) and fully unfolded state (State‐1) of the origami structure; (b,c) contour maps; (d–f) reflectance spectra; (g) variation in the center frequency of Peak 1 in State‐0 with rotation angle; (h) experimental and simulated center frequencies of Peak 2 in State‐0 as a function of rotation angle; (i–k) experimental and simulated center frequencies of Peak 1, Peak 2, and Peak 3 in State‐1 as a function of rotation angle; (l,m) reflectance amplitudes of Peak 1 and Peak 2 in State‐0 as a function of rotation angle; (n–p) reflectance amplitudes of Peak 1, Peak 2, and Peak 3 in State‐1 as a function of rotation angle.

Figure 6a–c shows the electric‐field distributions and power‐loss density distributions corresponding to the two resonance points at 3.74 and 17.05 GHz in State‐0. At 3.74 GHz, both the electric field and power loss are mainly concentrated in the lower main body, both shoulder regions, and edge regions of the T‐shaped SrTiO_3_ ceramic plate, whereas at 17.05 GHz, a more dispersed multiregion localized distribution is observed, indicating that the high‐frequency response involves electric‐field polarization and energy dissipation in multiple local regions of the ceramic plate. Figure 6d–g presents the electric‐field and power‐loss density distributions at the three resonance points of 3.26, 9.97, and 17.61 GHz in State‐1. Compared with State‐0, the low‐frequency response at 3.26 GHz after unfolding is redistributed from the lower region of the ceramic plate to the edge, corner, and head regions, corresponding to the redshift of the low‐frequency peak from 3.74 to 3.26 GHz. At 9.97 GHz, the electric field and power loss are mainly concentrated in the central and head regions of the ceramic plate, indicating that the mid‐frequency dielectric resonance response forms a clearer local excitation in the unfolded configuration. At 17.61 GHz, the field and loss distributions exhibit discrete localization in the edge, corner, and local internal regions, corresponding to the localized reconstruction of the high‐frequency response. The simulated reflectance curves in Figure 6h–j further show that, after switching from State‐0 to State‐1, the peak shifts and reflection‐valley depth variations in different frequency bands are generally consistent with the experimental trends shown in Figure 5d–f. These results indicate that the axial height variation and in‐plane torsion of the Kresling origami jointly modify the spatial position, in‐plane orientation, and propagation phase condition of the T‐shaped SrTiO_3_ ceramic plates, thereby reconstructing the local electric‐field and power‐loss distributions in different frequency bands and leading to frequency‐dependent modulation characterized by a low‐frequency redshift, mid‐frequency reflection‐valley enhancement, and high‐frequency blueshift.

**FIGURE 6:**
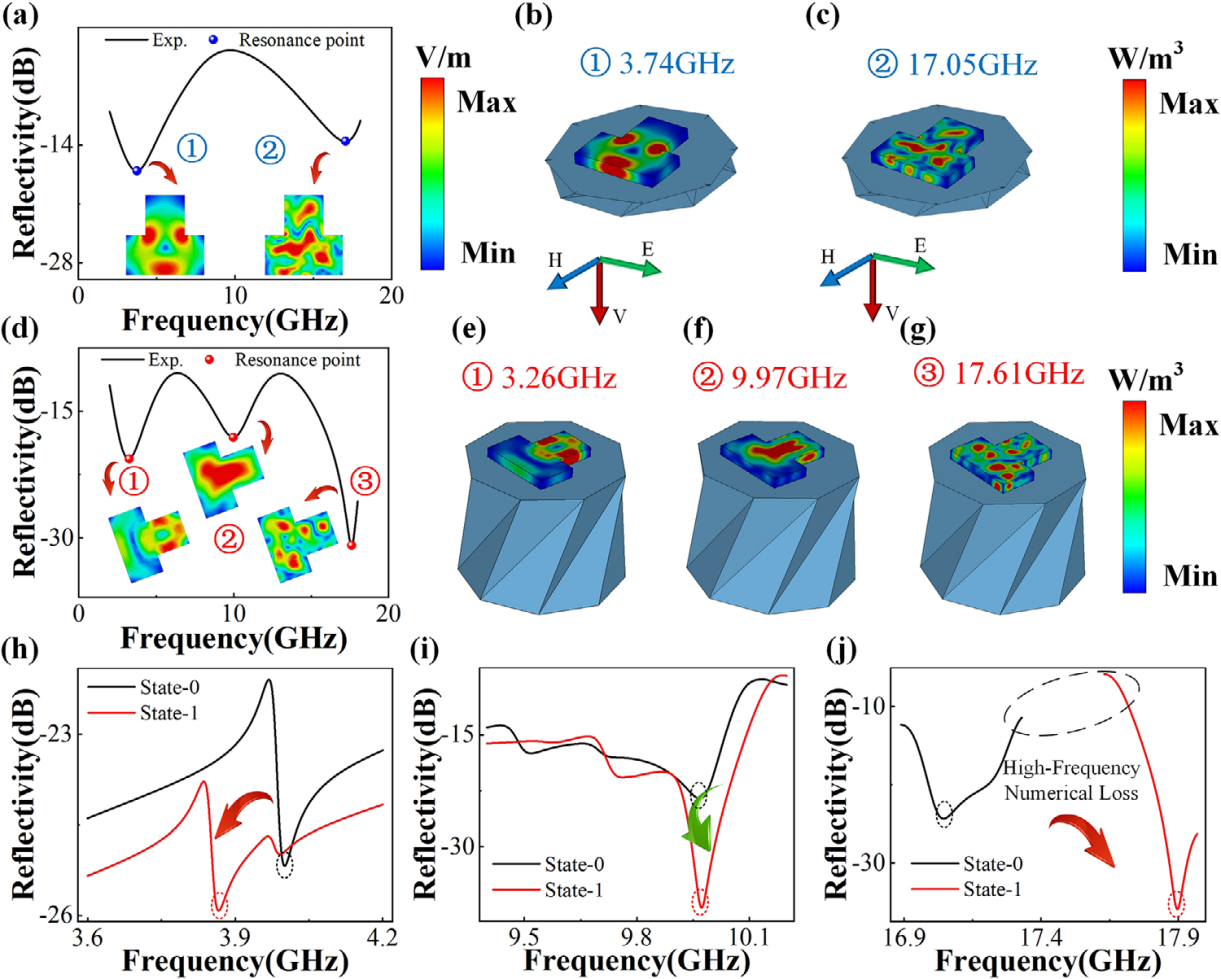
(a) Electric‐field distributions corresponding to the resonance points in State‐0; (b,c) loss distributions at 3.74 and 17.05 GHz; (d) electric‐field distributions corresponding to the resonance points in State‐1; (e–g) loss distributions at 3.26, 9.97, and 17.61 GHz; (h–j) simulated reflectance spectra during the transition from State‐0 to State‐1.

As shown in Figure 7a,e, in the co‐oriented arrangement of State‐0, the resonance modes exhibit good angular stability. As shown in Figure 7b,c and f,g, the center frequencies of Peak 1 and Peak 2 are stabilized within 3.71–3.87 GHz and 16.72–17.26 GHz, respectively, with corresponding minimum reflectance values of −20.6 dB and −24.1 dB. Figure 7d,h shows that the two resonance peaks follow highly consistent trends with the rotation angle, indicating that in‐phase coupling between units dominates in this configuration and that the overall electromagnetic response exhibits stable rotational invariance. When the ceramic arrangement is switched to the reversed configuration of State‐2, the system symmetry is altered, and the out‐of‐phase coupling effect is clearly enhanced, as schematically shown in Figure 7i,m. As shown in Figure 7j, the low‐frequency Peak 1 still maintains good angular stability, whereas the high‐frequency Peak 2 shown in Figure 7n exhibits strong angular fluctuations near 16.57 GHz. This phenomenon indicates that the high‐frequency resonance mode is more sensitive to phase‐interference effects induced by out‐of‐phase coupling, and its resonance state is more readily modulated by the relative orientation between units, thereby leading to a significant decrease in frequency stability. Figure 7k,o shows that the center frequencies of the two resonance peaks shift to 4.36–4.45 GHz and 16.31–16.72 GHz, respectively, showing clear frequency shifts. Figure 7l,p presents the variations in the reflectance amplitudes of the two resonance peaks in State‐2 with the rotation angle. Peak 1 shows reflection‐valley deepening around intermediate angles, with a relatively limited modulation amplitude, whereas Peak 2 exhibits more pronounced fluctuations and a larger reflectance modulation range. These results indicate that the arrangement direction of the T‐shaped SrTiO_3_ ceramic plates is an important structural parameter for tuning the phase relationship and local response differences among array units. The co‐oriented arrangement is favorable for maintaining a relatively stable dual‐peak response, whereas the reversed arrangement can introduce more pronounced orientation differences and out‐of‐phase coupling, thereby enabling resonance‐frequency shifts and reflectance‐amplitude modulation.

**FIGURE 7:**
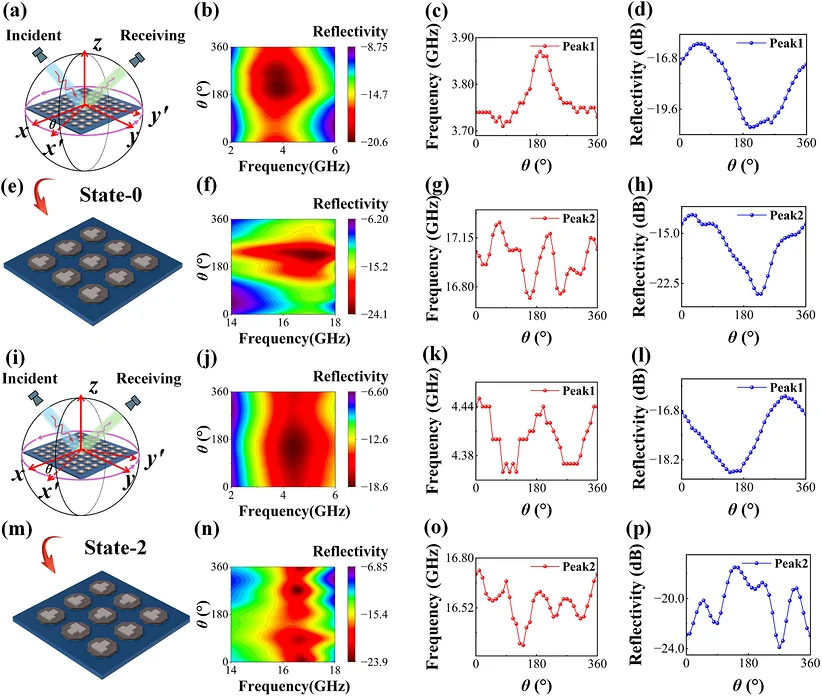
Modulation of electromagnetic waves by changing the arrangement of dielectric ceramic plates: (a,e) schematic diagrams of the co‐oriented SrTiO_3_ arrangement in State‐0; (b–d) contour map, center frequency, and reflectance amplitude of Peak 1 in State‐0 as a function of *θ*; (f–h) contour map, center frequency, and reflectance amplitude of Peak 2 in State‐0 as a function of *θ*; (i,m) schematic diagrams of the reversed SrTiO_3_ arrangement in State‐2; (j–l) contour map, center frequency, and reflectance amplitude of Peak 1 in State‐2 as a function of *θ*; (n–p) contour map, center frequency, and reflectance amplitude of Peak 2 in State‐2 as a function of *θ*.

Figure 8a,e,i shows the configuration design of the State‐3 spatially heterogeneous array and the corresponding rotation‐based measurement scheme. This configuration is formed by arranging folded and unfolded units in alternate columns, and during the reflectance measurement, the array rotates as a whole about the *z*‐axis by an angle of *θ*. The 2D reflectance contour map in Figure 8b shows that, within the 2–18 GHz frequency range, the system reflectance exhibits dynamic variations with the rotation angle *θ*, with values ranging from −9.24 dB to −54.4 dB. The low‐reflectance regions are mainly concentrated in the 10–15 GHz band, indicating that the spatially heterogeneous arrangement introduces an angle‐dependent electromagnetic response. Figure 8c presents representative reflectance spectra at *θ* = 60°, 120°, 180°, 240°, and 300°. As the rotation angle increases from 180° to 240° and then to 300°, the two mid‐to‐high‐frequency reflection valleys gradually evolve from spectral proximity and enhanced overlap to separated states, reflecting an interpeak coupling and decoupling process. Figure 8d compares this work with previously reported tunable metamaterial schemes. This work achieves a reflection modulation depth of 51.0 dB at approximately 12 GHz, highlighting the advantage of the spatially heterogeneous origami array for mid‐to‐high‐frequency microwave modulation with a large modulation depth. Figure 8f–h presents the center frequencies of Peak 1, Peak 2, and Peak 3 in State‐3 as functions of the rotation angle *θ*. Peak 1 remains within a narrow frequency range of 3.80–3.84 GHz, showing good frequency stability, whereas Peak 2 and Peak 3 are distributed within 10.97–12.54 GHz and 12.27–13.67 GHz, respectively, and exhibit more pronounced frequency shifts with rotation angle. Figure 8j–l shows the reflectance‐amplitude variations of the three resonance peaks. The minimum reflectance of Peak 1 is approximately −23.2 dB, whereas those of Peak 2 and Peak 3 reach −51.9 dB and −53.6 dB, respectively, indicating that the mid‐to‐high‐frequency responses are more sensitive to the spatially heterogeneous arrangement and rotation angle. The shaded regions in Figure 8g,h,k,l correspond to the spectral proximity, reflection‐valley deepening, and subsequent separation of Peak 2 and Peak 3 within specific rotation‐angle ranges, suggesting that the spatially heterogeneous array can introduce interpeak coupling through the height difference, orientation difference, and local response difference between folded and unfolded units. Figure 8m–p compares the electric‐field distributions of State‐3 at 11.9 GHz under different polarization conditions. Under TE polarization, the electric field is mainly concentrated on selected ceramic units and their edge regions, whereas under TM polarization, the electric‐field distribution is reconfigured and localized enhancement regions appear near ceramic units at different height levels. These results indicate that the State‐3 spatially heterogeneous arrangement can modify the local field distributions and interaction behavior of folded and unfolded units, which supports enhanced mid‐to‐high‐frequency dielectric resonance responses under specific rotation‐angle and polarization conditions, thereby providing a structural basis for programmable microwave reflectance modulation with a high modulation depth.

**FIGURE 8:**
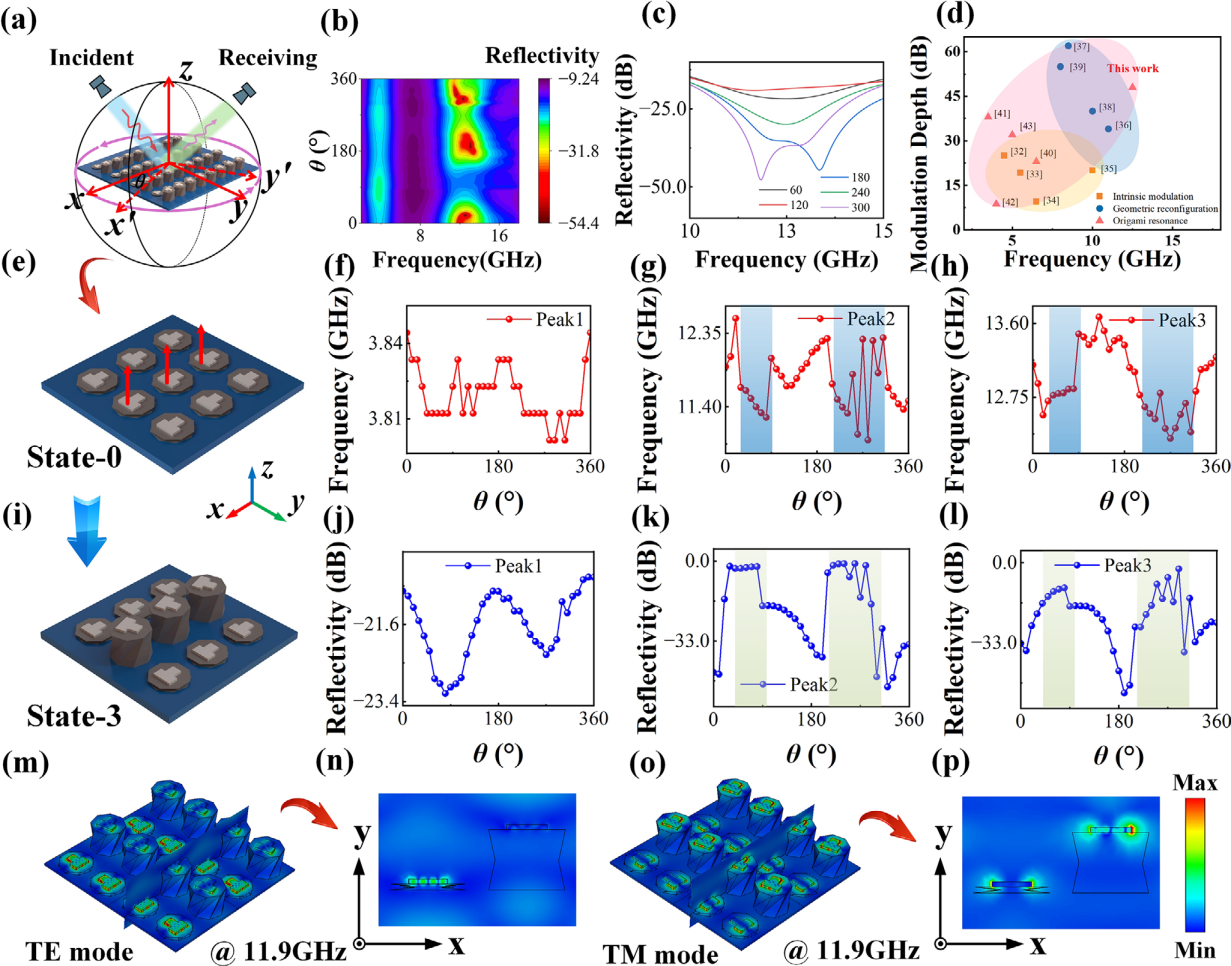
Modulation of electromagnetic waves by changing the origami structural arrangement: (a) schematic diagram of the State‐3 spatially heterogeneous array with folded and unfolded columns arranged alternately during rotation about the *z*‐axis; (b) contour map of reflectance as a function of frequency and rotation angle *θ* from 2 to 18 GHz; (c) reflectance spectra at *θ* = 60°, 120°, 180°, 240°, and 300°, showing coupling and decoupling behavior; (d) comparison of operating frequency and modulation depth between this work and existing tunable metamaterial schemes [32, 33, 34, 35, 36, 37, 38, 39, 40, 41, 42, 43]; (e) simplified structural schematic of State‐0; (f–h) center frequencies of Peak 1, Peak 2, and Peak 3 as functions of rotation angle *θ*; (i) simplified structural schematic of State‐3; (j–l) reflectance amplitudes of Peak 1, Peak 2, and Peak 3 as functions of rotation angle *θ*, where the shaded regions indicate interpeak coupling modulation; (m,n) cross‐sectional electric‐field distributions under TE polarization at 11.9 GHz; (o,p) cross‐sectional electric‐field distributions under TM polarization at 11.9 GHz.

## 4 Conclusion

This work establishes a direct, structure‐driven link between mechanical deformation and microwave resonance using bistable Kresling origami. Unlike electronic tuning schemes that demand continuous power or external biasing, our pneumatic metamaterial robot achieves spectral control through geometry alone. The coupled extension‐twisting motion of each unit tailors the axial height, orientation, phase, and boundary conditions of the integrated SrTiO_3_ patches. Single‐unit unfolding yields a resolvable reflection peak while rotating patches shift dual resonance centers across distinct bands. A heterogeneous array further enables coupled regulation, pushing reflectance to −54.4 dB with a modulation depth of 51.0 dB at 12 GHz. These results validate origami reconfiguration as a structural degree of freedom for programming notch number, frequency, and depth. The bistable design retains states without sustained power, overcoming key limitations of conventional tunable metamaterials and opening a pathway toward mechanically programmable microwave devices.

## Author Contributions

**Shaojie Xi**: data curation, investigation. **Xinxi Zeng**: methodology, investigation, supervision, funding acquisition, visualization, project administration, resources, writing – review and editing. **Long Zhou**: investigation, validation, writing – original draft, writing – review and editing. **Bo Li**: supervision, investigation. **Xian Wang**: data curation, supervision, formal analysis. **Jiang Luo**: investigation, data curation, resources, conceptualization. **Desheng Pan**: validation, funding acquisition, resources. **Haoxin Sun**: methodology, investigation, validation. **Lingyun Qian**: investigation, data curation, supervision. **Peipei Li**: data curation, supervision, investigation. **Chaoyang Sun**: investigation, methodology, project administration. **Yixuan Gao**: supervision, data curation, investigation. **Ji Zhou**: formal analysis, supervision, validation, investigation. **Pengfei Zhu**: investigation, validation, supervision.

## Funding

The National Natural Science Foundation of China: 52302132, 51788104, 51532004 and 52305334; The Ninth Research Institute of China Electronics Technology Group Corporation's open projects: 2024SK‐003‐2; The Beijing Natural Science Foundation: L223029; The Foshan Municipal People's Government Special Fund Project for Science and Technology Innovation: BK22BE017; The National Key Research and Development Program of China: 2023YFB3506700; the Interdisciplinary Research Project for Young Teachers of USTB (Fundamental Research Funds for the Central Universities): FRF‐IDRY‐24‐007.

## Conflicts of Interest

The authors declare no conflicts of interest.

## Data Availability

The data that support the findings of this study are available from the corresponding author upon reasonable request.
